# MicroRNA-421 Dysregulation is Associated with Tetralogy of Fallot

**DOI:** 10.3390/cells3030713

**Published:** 2014-07-11

**Authors:** Douglas C. Bittel, Nataliya Kibiryeva, Jennifer A. Marshall, James E. O’Brien

**Affiliations:** Ward Family Heart Center, Children’s Mercy Hospitals and Clinics and University of Missouri-Kansas City School of Medicine, 2401 Gillham Rd. Kansas City, MO 64108, USA; E-Mails: nkibiryeva@cmh.edu (N.K.); jmarshall@cmh.edu (J.A.M.); jobrien@cmh.edu (J.E.O.)

**Keywords:** tetralogy of Fallot (TOF), cardiac development, microRNA, miRNA, miR-421, SOX4, Notch signaling

## Abstract

The importance of microRNAs for maintaining stability in the developing vertebrate heart has recently become apparent. In addition, there is a growing appreciation for the significance of microRNAs in developmental pathology, including the formation of congenital heart defects. We examined the expression of microRNAs in right ventricular (RV) myocardium from infants with idiopathic tetralogy of Fallot (TOF, without a 22q11.2 deletion), and found 61 microRNAs to be significantly changed in expression in myocardium from children with TOF compared to normally developing comparison subjects (O’Brien *et al.* 2012). Predicted targets of microRNAs with altered expression were enriched for gene networks that regulate cardiac development. We previously derived a list of 229 genes known to be critical to heart development, and found 44 had significantly changed expression in TOF myocardium relative to normally developing myocardium. These 44 genes had significant negative correlations with 33 microRNAs, each of which also had significantly changed expression. Here, we focus on miR-421, as it is significantly upregulated in RV tissue from infants with TOF; is predicted to interact with multiple members of cardiovascular regulatory pathways; and has been shown to regulate cell proliferation. We knocked down, and over expressed miR-421 in primary cells derived from the RV of infants with TOF, and infants with normally developing hearts, respectively. We found a significant inverse correlation between the expression of miR-421 and SOX4, a key regulator of the Notch pathway, which has been shown to be important for the cardiac outflow track. These findings suggest that the dysregulation of miR-421 warrants further investigation as a potential contributor to tetralogy of Fallot.

## 1. Introduction

The heart is the first organ to form during mammalian embryogenesis and is critical for the viability of the embryo. Complex communication between cells and tissue is required to ensure the correct specification of cell lineage necessary for embryological heart formation [1]. Failure of proper cellular differentiation, migration and/or apoptosis results in congenital heart disease (CHD). CHD is a major cause of childhood morbidity and mortality, and remains a substantial challenge even in countries with advanced health care systems. Heart defects are the most common birth defect, occurring in approximately eight per 1000 live births [2]. Mendelian and chromosomal syndromes account for about 20% of all cases of CHD. The remaining 80% of CHDs are “sporadic”, and have proven intractable to genetic investigation. These sporadic events are most often inherited from unaffected parents, suggesting incomplete penetrance [3]. Variable penetrance can be explained, at least in part, by differences in the genetic buffering capacity between individuals [4,5]. 

Tetralogy of Fallot (TOF) is one of the more common severe forms of CHD with an incidence estimated at five to seven per 10,000 live births, thus representing 5%–7% of all congenital heart lesions. TOF is characterized by a misalignment of the conal septum leading to a rightward deviation of the aorta. This results in a large ventricular septal defect (VSD) and varying degrees of right ventricular outflow tract narrowing. TOF generally requires surgical repair in the first year of life.

MicroRNAs are important posttranscriptional inhibitors of gene expression resulting from the degradation of target mRNA or inhibition of translation. A powerful aspect of microRNA function is the ability of individual microRNAs to coordinate and regulate multiple target genes encoding proteins with related functions (e.g., stem cell differentiation, neurogenesis, skeletal and cardiac muscle development and function) [6]. Furthermore, individual mRNAs can be targeted by multiple microRNAs, allowing for enormous combinatorial complexity and regulatory potential. 

Recently, it has become apparent that mammalian cardiac development depends on the correct spatiotemporal expression of specific microRNAs [7]. For example, miR-1 promotes embryonic stem cell differentiation toward a cardiomyocyte lineage, whereas miR-133 inhibits differentiation into cardiac muscle. MiR-1 targets the Notch ligand Delta-like-1 (Dll-1) [8] and the transcription factor Hand2 [9] which is required for right ventricular growth and cardiomyocyte expansion. Furthermore, miR-1 knockout mice had thickened ventricles resulting from hyperplasia. Deletion of miR-1-2 causes lethal VSDs [10]. MiR-133a-1/133a-2 double knockout in mice results in inappropriate expression of SRF and cyclin D2 leading to late embryonic neonatal lethality due to VSDs and chamber dilatation [11]. Deletion of either miR-106b-25 or miR-106a-363, combined with the miR-17-92 null allele, can result in embryonic lethality accompanied by severe VSDs, atrial septal defects, and thin walled myocardium [12]. 

A few miRNAs have been associated with CHDs. Kuhn *et al.* reported that five human chromosome 21-derived microRNAs (miR-99a, let-7c, miR-125b-2, miR-155, and miR-802) are overexpressed in hearts from subjects with Down syndrome and CHD [13]. Also, miR-196a, which is an upstream regulator of Hoxb8 and Sonic hedgehog (Shh), has been associated with sporadic CHD [14]. We recently evaluated microRNA expression in right ventricular tissue from children with tetralogy of Fallot compared to tissue from normally developing age-matched comparison subjects [15]. We found multiple microRNAs with altered expression, accompanied by an inverse expression pattern of many genes predicted to be targeted by these microRNAs. In addition, we evaluated gene expression patterns in the RV of infants with TOF and found a significant suppression of both the Notch and Wnt pathways relative to normally developing tissue [16]. Intriguingly, many of the microRNAs we identified with altered expression in the RV of infants with TOF are predicted to interact with genes in the Notch or Wnt pathways. Of particular interest was SOX4, predicted to be targeted by several of the microRNAs and shown to interact with several key developmental pathways including the Notch and Wnt pathways [17]. SOX4 has been shown to be essential for cardiac outflow track formation [18,19]. Taken together, these studies support a significant role for microRNAs in mammalian heart development. Never the less, the extent and mechanism by which microRNAs contribute to human CHD remains poorly investigated. 

In our previous study, the microRNA with greatest change in expression was miR-421. *In silico* analysis of the potential targets of miR-421 predict it will interact with multiple genes known to regulate heart development, most interestingly SOX4. Furthermore, miR-421 has recently been identified in multiple forms of cancer, apparently playing a role in cell proliferation [20,21]. Here we present evidence that miR-421 does indeed modulate the expression of genes of importance to heart development and therefore, could play a role in congenital heart defects.

## 2. Experimental Section

### 2.1. Subjects

Our subjects were children less than one year of age with tetralogy of Fallot (TOF) requiring surgical reconstruction. The diagnosis and anatomy were obtained by echocardiography and angiography, and confirmed at the time of surgery. Informed consent was obtained from a parent or legal guardian after reviewing the consent document and having their questions answered. All proper institutional review board approvals were obtained for this study. Microarray analyses were run on samples from 16 infants with nonsyndromic TOF (*i.e.*, no 22q11.2 deletion) and eight infants with normally developing hearts (Table 1). Comparison tissues from eight normally developing infants (three males, five females) were obtained from LifeNet Health [22]. The control subjects were matched for age to the study population, and all control subjects expired due to non-cardiac related causes. The acquisition and pathology of these samples was described in detail previously [15].

**Table 1:** Age and Sex of subjects.

	n	Gender	Mean Age (Range)	Analyzed by Array	Analyzed by qRT-PCR
TOF	16	11M/5F	276 days (98–510)	16	16
control	8	3M/5F	142 days (28–382)	8	8
additional TOF subjects used for validation	8	4M/4F	292 days (167–425)	0	8

### 2.2. Cell Cultures and Transfection

Primary cells from infants with TOF were derived from right ventricular tissue removed at the time of surgical repair of the conotruncal outflow track. The RV was minced in DMEM (Life Technologies, Grand Island, NY) plus 10% fetal calf serum and 1% penn/strep (Life Technologies), and placed in an incubator with 5% CO_2_. After cells were established, they were trypsinized and grown in T-75 flasks with 10%/1% DMEM. We obtained a primary neonatal cardiomyocyte cell culture derived from normally developing human neonatal cardiac tissue from Celprogen (Celprogen Inc., Torrence, CA, Cat#36044-21). These cells were grown in the same media as the TOF primary cells. All transfections on primary cells were performed three times, each on a different cell line (genotype) and expression changes were normalized and used for analysis. Transfections on the primary cells from the normally developing infant heart were repeated three times.

**Figure 1:**
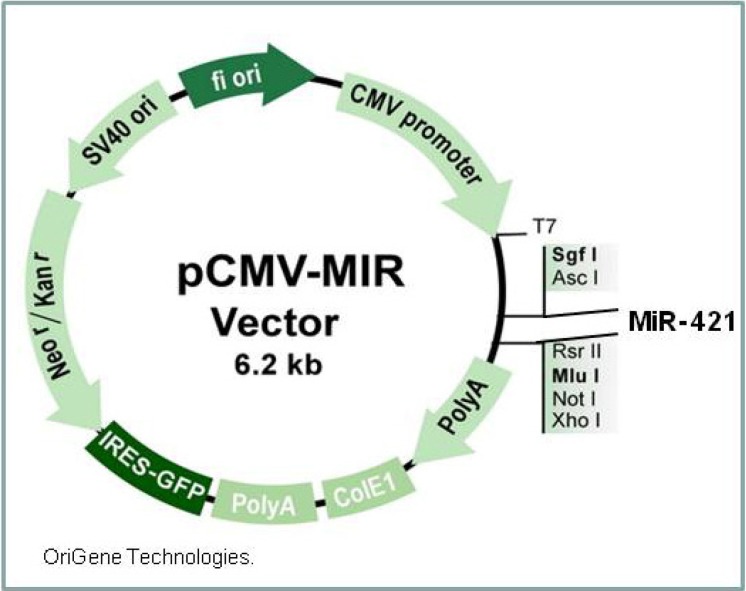
MiR-421 expression vector.

The miR-421 expression vector was ordered from Origene (Origene Technologies, Rockville MD, Figure 1, MIR421, cat #MI0003685,). We sequenced the plasmid after amplification to ensure that the miR-421 sequence was intact. Plasmid transfections were done using PolyMag transfection reagent according to the manufacturer’s instructions (Oz Bioscience Inc., San Diego, CA, USA). Briefly, 2 μg plasmid DNA was diluted in 200 μL of serum/supplement free media and added to 8 μL PolyMag transfection reagent, vortexed and incubated for 20 min at room temperature. The transfection mixture was added dropwise to 3 × 10^5^ cells in 1.8 mL of serum containing media in a single well of a 6 well plate. The plate was set on top of a plate magnet (Oz Biosciences) for 20 min, and returned to the incubator. After 72 h, the cells were trypsinized, pelleted and stored at −80 °C until processed for RNA analysis. 

We ordered flexitube antisense siRNAs for knockdown experiments targeting miR-421 (Qiagen Inc., Valencia, CA, Cat # Hs_MIR421-1). Knockdown experiments were done using HiPerfect transfection reagent (Qiagen, Valencia, CA) following the manufacturer’s instructions. Briefly, 50 μM siRNA diluted in 100 μL serum free media was mixed with 12 μL HiPerfect transfection reagent and incubated for 20 min at room temperature. The transfection mixture was added to 2 × 10^5^ cells in 2.3 mL media with 10% serum in a single well of a 6 well cell culture plate. After 72 h, the cells were pelleted and stored at −80 °C until processing. 

### 2.3. RNA Preparation

RNA was extracted from ~10 mg of frozen tissue from the right ventricle or cells using a mirVana microRNA isolation kit (Applied Biosystems/Ambion, Austin, TX, USA) according to manufacturer’s instructions. The control tissues (cryo-preserved pulmonary homografts) were thawed per protocol in sterile conditions. Tissue samples obtained during patient surgery (attending surgeon JEO) were immediately de-identified and frozen. All tissue samples removed during surgery were excised by the performing surgeon for clinical indications utilizing standard of care procedures. While a subset of patients were previously palliated with a modified Blalock-Taussig (BT) shunt, the right ventricular outflow tract region from which the tissue was harvested had not undergone any previous surgical manipulation. One author (JEO) aseptically dissected samples from the normally developing control heart tissue to ensure the tissues were from equivalent locations.

### 2.4. Microarray Analysis

The microRNA microarrays were Affymetrix GeneChip microRNA-1.0 (Affymetrix Inc. Santa Clara, CA, USA). The GeneChip microRNA-1.0 array contains probes for 847 human microRNAs and 922 human snoRNAs (the snoRNAs are inclusive of scaRNAs). The exon arrays were Affymetrix HuEx-1_0-st-v2. The raw data for the microRNA arrays have been deposited in the Gene Expression Omnibus (microRNA arrays -accession number GSE35490). All arrays were run at the Kansas University Medical Center-Microarray Facility (KUMC-MF) according to the manufacturer’s protocols. The KUMC-MF is supported by the Kansas University-School of Medicine, KUMC Biotechnology Support Facility, the Smith Intellectual and Developmental Disabilities Research Center (HD02528), and the Kansas IDeA Network of Biomedical Research Excellence (RR016475). 

All statistical analyses were performed using statistical software: Partek Genomics Suite software version 6.6 (Partek Inc, St. Louis, MO, USA), and Ingenuity Pathways (Ingenuity systems, Inc. Redwood City, CA, USA) as described previously [15]. Raw data (CEL. files) were uploaded into Partek Genomics Suite for normalization and statistical analysis. Robust Multichip Analysis (RMA) was used for background correction followed by quantile normalization with baseline transformation to the median of the control samples. Only probes with intensity values above 20% of background value, in at least one of the conditions, were included for additional analysis. A Student t-test with a Benjamini and Hochberg multiple test correction for false discovery rate (FDR) was used to determine significance. Probes were filtered using an FDR-adjusted *p*- value ≤ 0.05. 

The Ingenuity Pathways Analysis (IPA) version 9.0 (Ingenuity Systems, Inc., Redwood City, CA, USA) was used to explore networks, canonical pathways, predefined functional categories, IPA compiles experimentally validated miRNA/mRNA interactions as well as providing predicted microRNA/mRNA interactions using integrated access to TargetScan (Human), version 5; TarBase and MiRecords. Predicted interactions are scored as highly probable or moderately probable. We limited our miRNA target assessment to highly probable interactions only. IPA contains hundreds of pathways and identifies significant associations between the experimental data set and canonical pathways, functional categories or disease associations within the database by comparing the ratio of the number of molecules from the dataset that map to a given pathway, divided by the total number of molecules that map to the pathway. A Fisher’s exact test was used to calculate a *p*-value determining the probability that the association between the genes in the dataset and the pathway was explained by chance alone. All biological functions and/or diseases in IPA’s database were considered for the analysis without bias. Significance was defined as a *p*-value ≤ 0.05.

### 2.5. Real-Time Quantitative Polymerase Chain Reaction

To validate gene expression, quantitative reverse transcription-PCR (qRT-PCR) was performed on a subset of genes/transcripts using Taqman assays (Applied Biosystems. Inc., Life Technologies, Grand Island, NY, USA) according to the manufacturer’s instructions. Briefly, 2 ng of total RNA from each sample was reversed-transcribed using a microRNA specific Taqman microRNA reverse transcription kit. For each sample, real-time qRT-PCR was performed in triplicate on an ABI 7000 sequence detection instrument. The point at which the intensity level crossed the PCR cycle threshold (C_T_) was used to compare individual reactions. *RNU24* and *GAPDH* were used for normalization using the standard ^ΔΔ^C_T_ method. Results were calculated as fold change relative to control subjects.

## 3. Results and Discussion

We previously reported that the right ventricle of children with TOF had significantly altered expression of 61 microRNAs compared to the RV of normally developing infants [15]. We used Ingenuity Pathway Analysis to identify highly probable mRNA targets of the 61 microRNAs with altered expression. We then limited the potential target genes to a list of genes that we previously identified as important for cardiac development [15,23]. We then eliminated genes from the list that did not have significant changes in expression between the RV from infants with TOF and normally developing infants. We used this final list of filtered genes to create an interconnected network of cardiac genes and microRNAs with altered expression in TOF, all with a significant inverse correlation between expression level of the microRNA and the target mRNA (see Figure 1 in [15]). Thus, these microRNAs potentially regulate the expression of these genes and represent candidate microRNAs that contribute to TOF.

### 3.1. MiR-421 Expression is Increased in the RV of Infants with TOF.

**Figure 2:**
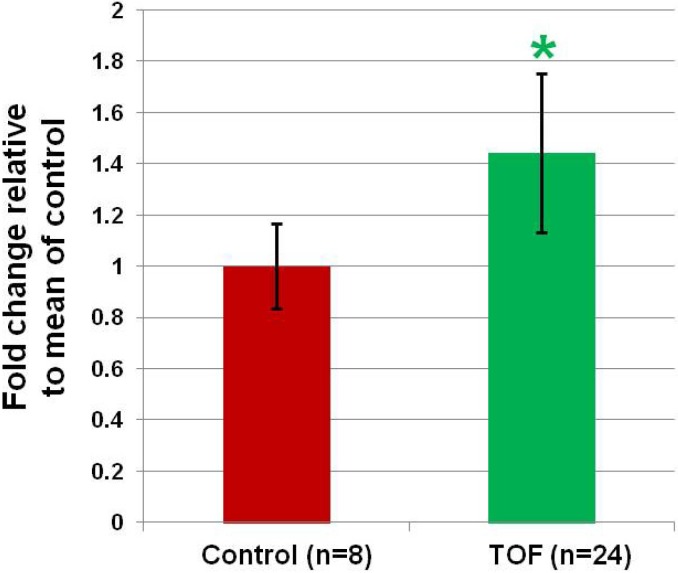
MiR-421 is more highly expressed in RV from infants with TOF compared to the RV from normally developing infants. Data is from qRT-PCR. * significant difference (*p* = 0.03).

Of the list of microRNAs, miR-421 had the greatest change in expression, increasing by 1.45 fold in the RV tissue from infants with TOF (Figure 2, *p* < 0.03). In addition, miR-421 is predicted to interact with several genes known to be critical for regulating heart development. We chose to focus on miR-421 for these reasons. 

**Figure 3:**
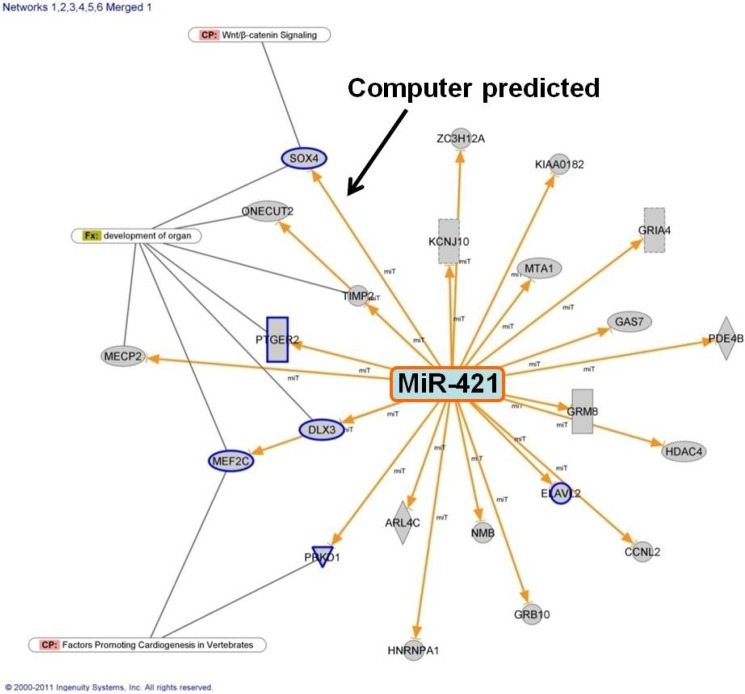
Putative connections between miR-421 and genes associated with cardiac development or function with significant inverse correlations between expression in TOF RV relative to control RV. Genes with a blue outline are members of the 5 key cardiac regulatory pathways shown in Figure 1 in [15].

We identified potential targets of miR-421 and created a network of genes that might be regulated by miR-421 (Figure 3). Multiple genes in the cardiac developmental network were predicted to interact with, or experimentally shown to interact with miR-421 (genes in blue outline, Figure 3). One of the genes predicted to be regulated by miR-421 is SOX4 which was significantly downregulated in our exon arrays using RNA from the RV of infants with TOF. We then investigated the potential interaction between miR-421 and SOX4, as SOX4 is a key regulator of the Notch and Wnt pathways [17] which regulate heart development. 

### 3.2. MiR-421 Expression is Inversely Correlated with SOX4 Expression

We compared the expression of multiple genes and microRNAs (including miR-421) by qRT-PCR between primary cells derived from the RV of infants with TOF and primary cardiomyocytes from normally developing infants. The expression of all microRNAs and genes examined retained the same relative expression levels between cell lines, as had been the case for the tissue examined. For example, the expression of miR-421 was increased in the cell lines derived from the RV of infants with TOF relative to the cells from normally developing infants (data not shown), as was the case for the tissue level comparisons. 

**Figure 4:**
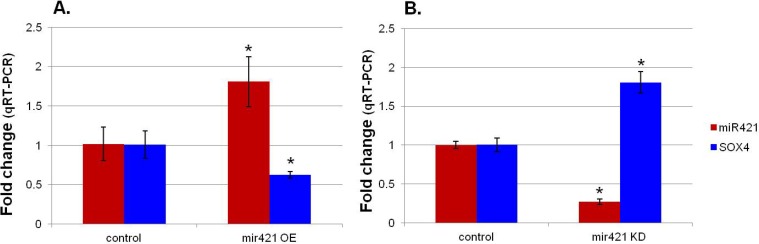
Transfections: (**A**) overexpression (OE) miR-421 in normal cardiomyocytes. (**B**) siRNA knockdown (KD) of miR-421 in primary cell cultures derived from the RV of infants with TOF. * significant change relative to starting level.

We transfected the primary cells from normally developing infants with the miR-421 expression vector (Figure 1) to increase expression of miR-421 in these cells. The transfected cells had a 1.8 fold increase (*p* = 0.023) in expression of miR-421 when 2 μg of the plasmid were used for transfection relative to nontransfected, or nonsense oligo transfected cells (Figure 4A and Table 2). 

Concurrently, the expression of SOX4 was reduced to 63% of its original level (*p* = 0.021, Figure 4A and Table 2). In addition, the reduction of SOX4 was dose responsive, decreasing as more of the expression vector was transfected into the cells and expression of miR-421 increased (data not shown).

**Table 2:** SOX4 response to changing levels of miR-421.

Sample Name	miR-421	SOX4
CP neo cells	1.02 ± 0.21	1.01 ± 0.18
miR-421 OE	1.81 ± 0.32 *	0.63 ± 0.04 *
*p* value	0.023	0.021
TOF cells	1.00 ± 0.05	1.00 ± 0.09
miR-421 KD	0.27 ± 0.03 *	1.81 ± 0.14 *
*p* value	0.004	0.011

CP neo = Celprogen neonatal cardiomyocytes from normally developing subjects; TOF cells = primary cells derived from the right ventricle of children with tetralogy of Fallot; OE = over expression; KD = knockdown * = significant change relative to starting level.

We used an antisense miR-421 siRNA to knockdown miR-421 expression in the primary cells derived from TOF tissue. When targeted by the antisense siRNA, miR-421 expression was reduced to 27% of its control level (*p* = 0.004, Figure 4B and Table 2). The expression of SOX4 consequently increased 1.8 fold (*p* = 0.011, Figure 4B and Table 2). Taken together, these data validate that miR-421 inversely influences the expression of SOX4. Since SOX4 has been shown to regulate the Notch and Wnt pathways [17], miR-421 may have played a role in the general suppression of the Notch and Wnt pathways that we previously observed [16].

## 4. Conclusions

MiR-421 is more highly expressed in the right ventricle of infants with tetralogy of Fallot than in the RV of infants with normally developing hearts. MiR-421 is predicted to interact with several genes that are important for heart development, and in the RV tissues there is an inverse correlation between the expression of miR-421 and SOX4, a key regulator of the Notch and Wnt pathways. We showed that altering the expression of miR-421 in primary cells derived from infant heart tissue has an inverse impact on the expression of SOX4. These data support a role for miR-421 in regulating SOX4, and potentially the Notch and Wnt pathways. However, miR-421 has multiple potential downstream targets and some of these may have indirectly altered the expression of SOX4. Thus, we are unable to conclude that there exists a direct inverse relationship between miR-421 and SOX4 based upon these experiments alone. However, our experiments confirm that miR-421 is overexpressed in RV tissue from infants with TOF and that SOX4 expression is responsive to miR-421 level. Further investigation of the role played by miR-421 in regulating the WNT and Notch pathways and heart development is justified. Finally, miR-421 should be added to the growing list of candidate genes that regulate heart development and when dysregulated may contribute to congenital heart defects. 

## Abbreviations

CHD: congenital heart disease
FDR: false discovery rate
ncRNA: noncoding RNA
qRT-PCR: quantitative reverse transcription-PCR
scaRNAs: small cajal associated RNAs
snRNA: small nuclear RNA
snoRNA: small nucleolar RNA
TOF: tetralogy of Fallot
VSD: ventricular septal defect
